# Nearly Complete Genome Sequences of Human Norovirus Belonging to Several Genotypes from Valencia, Spain

**DOI:** 10.1128/MRA.00641-19

**Published:** 2019-08-22

**Authors:** Cristina Santiso-Bellón, Carolina Monzó, Azahara Fuentes-Trillo, Susana Vila-Vicent, Juliana da Silva Ribeiro de Andrade, Roberto Gozalbo-Rovira, Javier Buesa, Felipe J. Chaves, Jesús Rodríguez-Díaz

**Affiliations:** a Department of Microbiology, School of Medicine, University of Valencia, Valencia, Spain; b Genomic and Genetic Diagnosis Unit, Hospital Clínico Research Foundation (INCLIVA), Valencia, Spain; c Laboratory of Comparative and Environmental Virology, Oswaldo Cruz Institute, Rio de Janeiro, Brazil; d CIBER of Diabetes and Associated Metabolic Diseases (CIBERDEM), Madrid, Spain; KU Leuven

## Abstract

Human noroviruses are responsible for most nonbacterial acute gastroenteritis cases. The GII.2, GII.4, and GII.17 genotypes of human noroviruses have recently arisen as the most frequent genotypes found in humans worldwide. We report here seven nearly complete genomes of these genotypes from patients with acute gastroenteritis in Valencia, Spain.

## ANNOUNCEMENT

Human noroviruses are responsible for 18% of all cases of acute gastroenteritis worldwide (1) and are the third most common mortality cause in children under 5 years old (2). The *Norovirus* genus belongs to the *Caliciviridae* family and is classified into six distinct genogroups (GI to GVI), which are further subdivided into different genotypes (3). Norovirus strains are subjected to continuous processes of punctual mutations and/or recombination. Therefore, new strains emerge periodically (4, 5). In the last few years, a new recombinant strain has emerged (GII.P4.NewOrleans2009_GII.4.Sydney2012). Together with this variant, GII.17 and GII.2 are responsible for the majority of outbreaks worldwide (6, 7).

We report here seven nearly complete genome sequences of human GII noroviruses (GII.2, GII.4, and GII.17) initially detected by conventional reverse transcriptase PCR (RT-PCR) in feces collected from both adults and children. Samples were from sporadic cases and outbreaks of acute gastroenteritis from November 2015 to September 2017. All patients were treated in different hospitals in Valencia, Spain.

Viral RNA was extracted from 250 μl of 10% fecal suspension in phosphate-buffered saline using TRIzol reagent (Invitrogen). Total RNA was sent to Macrogen, Inc., for library construction using a TruSeq mRNA library prep kit (Illumina) and sequencing on a HiSeq platform (Illumina). Raw read quality control was assessed with FastQC version 0.11.5 (8). Reads were quality trimmed using seqtk trimfq version 1.2-r101-dirty (9), and genomes were assembled using SPAdes version 3.11.1 (10) and MEGAHIT version 1.2.4 (11). The obtained contigs were annotated using BLASTn against the full viral genome database from NCBI. Contigs assigned as norovirus were further genotyped using the norovirus genotyping tool (12). All genome sequences contained three open reading frames (ORFs).

Table 1 summarizes the most relevant information of each of the sequences. The isolate 3453 sequence had a 99.22% (7,468/7,527 [number of nucleotides identical to the reference sequence compared to the total number of nucleotides of the obtained sequence]) nucleotide similarity with the isolate NORO_187_01_10_2015 GII.2 strain (MH218655). The isolate 3351 sequence had a 99.63% (7,505/7,533) nucleotide similarity with the Hu/GII.P17_GII.17/KR/2014/CAU-265 strain (KU561253). The isolate 3587 sequence shared 99.5% (7,520/7,558) nucleotide similarity with the Hu/GII.17/142700/Shanghai/2014 strain (KT380915). The sequence of sample 3618 had a 99.11% (7,475/7,542) nucleotide similarity with the Hu/GII.P17_GII.17/KR/2014/CAU-265 strain (KU561253). The isolate 3546 sequence had a 98.55% (7,430/7,539) nucleotide similarity with the GII.P4 New Orleans 2009_GII.4 Sydney 2012 GII isolate NORO_141_02_11_2014 (MH218612). The sequence of sample 3549 shared a 99.55% (7,512/7,546) nucleotide similarity with the GII.Pe_GII.4 Sydney 2012 NORO_42_01_09_2014 strain (MH218701). The isolate 3552 sequence had a 98.45% (7,419/7,536) nucleotide similarity with the GII.P4 New Orleans 2009_GII.4 Sydney 2012 NORO_141_02_11_2014 strain (MH218612).

**TABLE 1:** Assembly characteristics for the norovirus contigs obtained^*a*^

Isolate	GenBank accession no.	Polymerase genotype^*b*^	Capsid genotype^*b*^	ORF1 nt^*c*^	ORF2 nt^*c*^	ORF3 nt^*c*^	No. of norovirus reads/raw reads (%)	Coverage (×)	Genome coverage (%) (genome length [bp])^*d*^	GC content (%)	GenBank accession no. of most similar strain (nt lacking in the 5′ end)
3453	MK789430	GII.P2	GII.2	1–5094	5075–6703	6703–7482	4,529,781/31,181,056 (14.5)	2,675.12	99.9 (7,536/7,546)	49.8	MH218655 (GTGAATGAAG)
3351	MK789431	GII.P17	GII.17	1–5097	5078–6700	6700–7499	1,360,087/94,666,148 (1.4)	2,587.67	99.9 (7,533/7,549)	48.2	KU561253 (GTGAATGAAGATGGCG)
3587	MK789432	GII.P17	GII.17	5–5113	5094–6716	6716–7495	67,237,010/70,923,098 (94.8)	7,674.57	100 (7,558/7,558)	48.1	KT380915
3618	MK789433	GII.P17	GII.17	1–5099	5080–6702	6702–7481	15,904,593/46,226,582 (34.4)	6,316.85	99.8 (7,542/7,556)	48.2	KU561253 (GTGAATGAAGATGG)
3546	MK789434	GII.P4 New Orleans 2009	GII.4 Sydney 2012	1–5093	5074–6696	6696–7502	39,211,847/90,843,600 (46.1)	7,716.42	99.9 (7,540/7,551)	49.9	MH218612 (GTGAATGAAGA)
3549	MK789435	GII.Pe	GII.4 Sydney 2012	5–5104	5085–6707	6707–7513	90,198,955/90,548,480 (99.6)	7,792.28	100 (7,569/7,569)	49.6	MH218701
3552	MK789436	GII.P4 New Orleans 2009	GII.4 Sydney 2012	1–5090	5071–6693	6693–7499	68,287,930/88,189,676 (77)	7,755.68	99.8 (7,536/7,550)	49.8	MH218612 (GTGAATGAAGATGG)

a nt, nucleotides.

b Polymerase and capside genotypes were assigned using the norovirus genotyping tool (12).

c The ORFs were automatically assigned by GenBank after sequence submission.

d The total expected genome length is determined relative to the sequence annotated as “full genome” with the higher similarity after BLAST search.

In summary, our study describes seven nearly complete genome sequences of different genotypes of norovirus isolates, providing additional important information to use as reference for phylogenetic and evolutionary studies.

### Data availability.

The GenBank accession numbers for the norovirus genomes are MK789430, MK789431, MK789432, MK789433, MK789434, MK789435, and MK789436. The sequence data are available in the Sequence Read Archive (SRA) under BioProject number PRJNA497363.
